# Resilient living program for patients with advanced cancer and their caregivers

**DOI:** 10.1017/S1478951524002128

**Published:** 2025-03-14

**Authors:** Sherry Chesak, Lori Rhudy, Susanne M. Cutshall, Konstantinos Leventakos, Cindy Tofthagen, Jay Mandrekar, Teresa A. Rummans, Matthew M. Clark, Shawna Ehlers, Maria I. Lapid, Amit Sood, Deirdre R. Pachman

**Affiliations:** 1 Division of Nursing Research, Mayo Clinic, Rochester, MN, USA; 2 Department of Graduate Nursing, Winona State University, Winona, MN, USA; 3 Division of Community Internal Medicine, Geriatrics and Palliative Care, Mayo Clinic, Rochester, MN, USA; 4 Department of Medical Oncology, Mayo Clinic, Rochester, MN, USA; 5 Division of Nursing Research, Mayo Clinic, Jacksonville, FL, USA; 6 Department of Quantitative Health Sciences, Mayo Clinic, Rochester, MN, USA; 7 Department of Psychiatry and Psychology, Mayo Clinic, Rochester, MN, USA; 8 Global Center for Resiliency and Wellbeing, Rochester MN, USA

**Keywords:** Advanced cancer, caregiver, resilience, anxiety, mindfulness

## Abstract

**Objectives:**

Patients with advanced cancer and their caregivers experience a substantial amount of anxiety and distress. The purpose of this study was to assess the feasibility, acceptability, and preliminary effects of an 8-week, remotely delivered Resilient Living Program (RLP) for adult patients with advanced cancer and their caregivers.

**Methods:**

Eligible patients included adults (≥18 years) with advanced cancer. Their caregiver had the option to participate. The RLP components included online modules, a print journal, and 4 video-telehealth-delivered sessions. Content focused on techniques for managing stress and building resilience (mindful presence, uplifting emotions, reframing experiences through practicing principles of gratitude, compassion, acceptance, meaning, and forgiveness). Feasibility and acceptability were assessed quantitatively and with semi-structured interviews conducted with a subset of participants. Effectiveness measures (anxiety, stress, quality of life [QOL], sleep, resiliency, and fatigue) were administered at baseline, week 5, week 9, and week 12.

**Results:**

Of the eligible patients, 33/72 (46%) were enrolled. In all, 15 caregivers enrolled. Thirty participants (21 patients/9 caregivers) completed at least 3 video-telehealth sessions (63% adherence). For patients, there were statistically significant improvements in anxiety and fatigue at week 12 (*p* = 0.05). Other effectiveness measures (stress, QOL, sleep, resiliency) showed positive trends. Eleven participants were interviewed and qualitative analysis revealed 4 themes: Easy to Use, Learning Key Principles, Practice is Essential, and Examples of Benefits.

**Significance of results:**

Participation in the RLP was feasible and acceptable for patients with advanced cancer and their caregivers. Participants tended to indicate that the practices were easy to integrate into their everyday lives, engendered their ability to focus on the positive, and would recommend the RLP to other individuals living with advanced cancer. Preliminary effectiveness data suggest the program may positively impact anxiety, stress, QOL, sleep, resiliency, and fatigue. A larger randomized clinical trial is warranted to confirm these preliminary findings.

## Introduction

Individuals living with advanced cancer experience a range of physical and psychological symptoms, psychosocial concerns, and existential angst that adversely impact their quality of life (QOL) (Moghaddam et al. 2016; Mollica et al. 2022; Vogt et al. 2021). Psychosocial interventions such as cognitive behavior therapy and supportive psychotherapy may be effective in reducing distress in these patients (Jacobsen and Jim 2008; Holland 2019). Mindfulness-based interventions have been studied to reduce cancer distress and have been found to improve QOL and decrease depression and anxiety. However, most of these studies are limited in that they included only patients with early-stage breast cancer and cancer survivors who completed cancer treatment (Zainal et al. 2013; Zhang et al. 2015; Huang et al. 2016; Haller et al. 2017; Rush and Sharma 2017). Patients with advanced cancer have unique psychosocial needs related to the incurable nature of their disease, ongoing burden of treatment, and prognostic uncertainty (Mollica et al. 2022). Therefore, it is essential to develop psychosocial interventions that specifically address the needs of this understudied population. The development and evaluation of interventions to improve coping and to address existential and psychosocial needs of individuals living with advanced cancers was identified as an area of need by the National Cancer Institute (Mollica et al. 2022). Moreover, it has been recognized that caregivers of individuals with advanced cancer suffer from significant emotional distress and high levels of burden and need interventions to address these issues.

There has been increasing research supporting the efficacy of interventions that address the needs for psychosocial support of both persons with chronic illness and their caregivers, demonstrating improvements in symptoms of depression and cancer-related distress (Milbury et al. 2020a, 2020b).

Overall, it has been shown that mindfulness-based interventions in patients with advanced cancer are acceptable and beneficial with improvements seen in QOL, enhanced acceptance of cancer diagnosis and treatments, and reduced symptoms of depression and anxiety (Zimmermann et al. 2018). However, most of these interventions differed in specifics of the studied intervention and utilized a range of outcome measures. Moreover, it was shown that mindfulness-based interventions are often limited by the time commitment required by the participant as patients with advanced cancer have limited energy and time (Zimmermann et al. 2018). This highlights the importance of developing and testing an easy to use and less time-intensive intervention. One such program is the Stress Management and Resiliency Training (SMART) program. The SMART program, developed by Dr Amit Sood, aims to train participants to develop intentional attention, experience more uplifting emotions, and reframe experiences through principles of gratitude, compassion, acceptance, meaning, and forgiveness. The core SMART training can be completed in about 2 hours, with daily practice of only 5–10 minutes. Studies have demonstrated the efficacy of the SMART program in improving resilience, perceived stress, anxiety, and overall quality of life in a variety of populations including breast cancer survivors (Loprinzi et al. 2011).

Given the unique psychosocial needs of patients living with advanced cancer and their caregivers, the SMART program was modified to create the Resilient Living Program (RLP) for patients with advanced cancer and their caregivers. The process of adapting the program involved input and review of a multidisciplinary team of providers with expertise in caring for patients with advanced cancer and their caregivers. This team partnered with Dr Amit Sood in making modifications that focused on relevancy to the population, including addressing the distinctive emotional and existential distress that the patient living with cancer and their caregiver experience individually and together. Further, the program was adapted to include online modules and remote facilitated sessions to make the program more accessible and decrease the burden of participation. These factors have been major limitations of prior studied interventions.

The purpose of this pilot study was to assess the feasibility and acceptability of the remotely delivered RLP for adult patients with advanced cancer and their caregivers. A secondary objective was to assess preliminary effects of the program on anxiety, stress, QOL, sleep, resiliency, and fatigue.

## Methods

### Study design and participants

A single-group pre/post study design was employed to assess outcomes of the RLP among advanced cancer patients and their caregivers. Inclusion criteria for both groups included age ≥ 18 years; English fluency; no diagnosed severe cognitive impairment; ability to provide written (paper or electronic) informed consent and complete questionnaires by themselves or with assistance; and the ability to utilize technology to watch online modules. Additional patient inclusion criteria included diagnosis of advanced, incurable solid tumor cancer; expected prognosis > 6 months, and baseline distress score ≥ 4/10 on the NCCN Distress Thermometer (DT) scale (0 = no distress; 10 = extreme distress) (Ma et al. 2014), or identified as having distress that would benefit from the program by the care team or provider. An additional inclusion criterion for caregivers was that they self-identified as a caregiver (broadly defined as family members, friends, or others who provide unpaid care) of a patient who also participated in the study. Caregivers were only able to participate in the study if the patient they cared for participated. However, patients could participate regardless of whether their caregiver chose to participate. Exclusion criteria, as determined through self-report, included those diagnosed with a history of a psychotic episode and/or other psychological co-morbidities such as untreated schizophrenia and bipolar disease. Potential participants were identified through the Palliative Care, Oncology, and Psychiatry and Psychology clinics at Mayo Clinic, in Rochester, MN. The study was approved by the Mayo Clinic IRB and consent was obtained from participants. The study is registered at clinicaltrials.gov.

### Intervention

The RLP is a psychosocial, mindfulness-based intervention that includes techniques for stress management and building resilience (mindfulness, uplifting emotions, reframing experiences through practicing principles of gratitude, compassion, acceptance, meaning, and forgiveness) (Table 1). For this study, participants were provided 4 individual sessions delivered via video telehealth. Sessions were 2 weeks apart and facilitated by trained interventionists (S.M.C., S.C., or D.R.P.). Interventionist training included a 6-month certified resilience training program offered by the Global Resilience and Inner Transformation Institute. The video sessions were delivered 1:1 with the interventionist and participant or with the interventionist and patient and caregiver together depending on their preference. Other intervention components included online video modules (www.resilientliving.net) developed and narrated by Dr Amit Sood. Participants were assigned to watch the modules on their own time over the 8-week intervention and were provided a companion journal with assignments in between sessions. Sessions were provided utilizing a Health Insurance Portability and Accountability Act-compliant real-time audio-visual connection video platform or telephone calls.

**Table 1.** Intervention components

**Introduction**	The Development of Resilience to Cope with Chronic Medical Conditions
	The Physiology of Stress Associated with Uncertain Life Circumstances
**Module I: Gratitude**	The Brain: A Back-Stage Tour
	Focus, Fatigue and Fear
	Attention Sumps
	Identifying What is Going Right within What is Going Wrong
	Creating a Gratitude Habit
	Morning Gratitude
	Grateful Memories
**Module II: Mindful Presence**	What is Mindful Presence?
	Three Domains of Attention
	Curious Moments, Scheduled Worry Time, 2-Minute Rule
**Module III: Kindness**	The Kindness Mortar
	Kind Attention, Creative with Kindness, Self-kindness
	Self-Kindness
	Kind Meditation
	The Two Brains
**Module IV: Resilient Mindset**	The Five Principles (Gratitude, Compassion, Acceptance, Meaning, Forgiveness)
	Practicing Self-Compassion
	Summary of Program
	Maintaining Ones Practice in Challenging Life Circumstances

### Study outcomes

Feasibility was measured by numbers and percentages of participant recruitment, enrollment, and retention of participants in the intervention. The enrollment target to satisfy feasibility was at least 25% of eligible patients and their caregivers enroll recognizing that not all patients will prefer to have their caregivers enroll. Eligible participants were defined as the number who fulfilled inclusion criteria and were approached to participate in the study. Adherence was defined as 75% of the enrolled participants completing at least 3 of the 4 remote video telehealth sessions. Acceptability was measured by an investigator-developed End-of-Study Questionnaire that was completed at week 9.

All participants were invited to participate in a semi-structured interview after their final facilitated remote video telehealth session to further assess feasibility and acceptability of RLP. Open-ended questions were used to assess the impact of the program: which principles of the program they practiced; the relevance of the program to an advanced cancer patient, or caregiver; if they would recommend the program to others in similar situations, their experiences with the various program delivery methods; and any suggestions for improvements. Patients who had a caregiver participate were offered the option to be interviewed individually or together. Interviews were conducted by an investigator, who did not facilitate the participants’ remote video intervention sessions via a web-based conferencing system. Interviews lasted up to 1 hour and were recorded and transcribed for analysis.

Quantitative effectiveness measures were administered at baseline, week 5 (after 2 remote video sessions), week 9 (end of intervention), and week 12 with valid and reliable instruments.

Concepts measured included anxiety (General Anxiety Disorder-7) (Spitzer et al. 2006), stress (Perceived Stress Scale) (Cohen et al. 1983), QOL (Linear Analog Self-assessment) (Bretscher et al. 1999), sleep (Insomnia Severity Index) (Bastien et al. 2001), resiliency (resiliency scale) (Southwick et al. 2014, 2015), and fatigue (Patient-Reported Outcomes Measurement Information System Fatigue Item Bank) (Cella et al. 2016). See Table 2 for instrument information.

**Table 2.** Instrument scoring, interpretation, and psychometrics

Instrument	Number of items	Scoring	Interpretation
NCCN Distress Thermometer	1	0–10	Higher scores = more distress
			Cut score of 4 = clinically meaningful distress
Linear Analog Self-assessment	6	0–10 on each item	Higher scores = better quality of life
Perceived Stress Scale	14	Positively stated items are reversed scored and all items’ scores are summed	Higher scores = more stress
Generalized Anxiety Disorder	7	Likert type scale with responses ranked from 0 to 3 for each item and summative scores ranging from 0 to 21	Higher scores = more anxiety Scores of 10–14 = moderate anxiety; 15–21 severe anxiety
Insomnia Severity Index	7	Likert type scale with responses ranked from 0 to 4 and summative scores from 0 to 28	Higher scores = more insomnia, scores > 15 indicate clinical insomnia
PROMIS Fatigue-Short Form	4	Likert type scale, responses ranging from 1 (never)- 5 (always) and total scores from 4 to 20	Higher scores = more fatigue
Resiliency Scale	10	0–10 on each item	Higher scores = higher resilience

### Data analysis

Data were entered into a REDCap data base. Continuous data were summarized as medians (minimum, maximum); categorical data were summarized as frequencies and percentages. The postintervention measurements were compared to baseline using Wilcoxon signed rank test due to smaller sample sizes and non-normally distributed outcomes. Analysis was performed separately for patients and caregivers. All tests were 2 sided and *p*-values less than 0.05 were considered statistically significant. Analysis was performed using SAS software version 9.4 (SAS Inc. Cary, NC).

Qualitative data were analyzed using content analysis (Graneheim and Lundman 2004; Lindgren et al. 2020). Transcripts were verified and read for overall impressions. A coding strategy was developed with independent coding by 2 research team members (L.R., S.C.); the coding strategy was verified by consensus. Exemplars supporting each theme are included to enhance credibility, confirmability, and transparency. An audit trail was maintained to ensure trustworthiness and confirmability. One of the researchers involved in the analysis did not conduct the interviews, which aided in minimizing bias. Data saturation (no new themes emerged) in later interviews.

## Results

### Feasibility/acceptability outcomes

Of the 72 eligible patients, 33 (46%) were enrolled; 15 also had a caregiver enrolled. Participant demographics are depicted in Table 3. A majority (63%) of participants completed at least 3 of the 4 remote sessions. Of the 33 patient participants, all completed baseline questionnaires and 12 completed the 12-week study questionnaires. Nine patients died within 9 months of study enrollment; other identified reasons for discontinuing participation included significant progression of their illness (5); fatigue (1), and travel (1); 5 provided no rational for study dropout. Of the 15 caregiver participants, all completed baseline questionnaires and 4 completed the 12-week study questionnaires.

**Table 3.** Demographics

Demographic	Patients (*n =* 33)	Caregivers (*n* = 15)
Age in years	28–87 (57.5)	24–81 (53.3)
Gender	Female: 22	Female: 10
	Male: 10	Male: 5
	Non-Binary: 1	
Race/Ethnicity	White: 31	White: 15
	African American: 1	
	Hispanic: 1	
Education		
<High School	1	0
High School	1	1
Technical or Associate	12	4
Bachelor’s Degree	9	3
Graduate Degree	9	7
Tumor type	Breast 7	
	Pancreatic 4	
	Colon 2	
	Lung 5	
	Mesothelioma 2	
	Gynecologic 6	
	Genitourinary 2	
	Other 5	
Relationship to patient		Spouse/Partner: 11
		Adult Child: 4

The End of Study Questionnaire results revealed that participants found the RLP helped decrease their stress level and enhanced their resilience (Table 4). Study participants noted the program was appropriate for their current life situation and overall felt that the number and length of sessions were acceptable. Those who participated with a caregiver found it to be a positive experience and felt that it improved their relationship. Participants reported that the online program and the video telehealth system for the facilitated remote sessions were easy to use; and they felt comfortable engaging with the interventionist via video.

**Table 4.** Program evaluation

Question	Mean Score** (*n* = 18)
Do you think the skills you learned helped decrease your stress level?	4.1
Do you think the skills you learned enhanced your resilience?	4.2
Would you recommend the program we shared with you to your friends and loved ones to help decrease their stress?	4.3
Did you feel the program was appropriate for your current life situation?	4.4
Did you feel like the number and length of sessions in the program was acceptable?	4.7
If you participated with your caregiver, did you find that to be a positive experience?	4.4
If you participated with your caregiver, do you feel like it improved your relationship?	4.0
Did you find the online program easy to use?	4.6
Did you have any difficulty completing the online program?	1.5
Were you able to watch all of the videos (1–50) in the online program?	Y:14 N:4
Did you find the journal easy to use?	4.3
Did you have any difficulty completing the journal entries? (1 = not at all; 5 = very much so)	2.7
I thought the video system used for virtual sessions with the investigators was easy to use.	4.8
I felt comfortable discussing my progress with the Resilient Living program by video with the investigators.	4.8
I felt comfortable discussing my progress with the Resilient Living program by phone with the investigators.	4.6

** Scores rage from (1 = not at all; 5 = very much so).

### Preliminary effects

Results of patient reported outcome measures are reported in Table 5. There was a consistent positive trend toward improvement in anxiety (GAD-7) across timepoints. For patients, at week 12 the improvement in anxiety was statistically significant (*p* = 0.05). There were also trends toward improvement in fatigue with statistically significant improvement at week 12 (*p* = 0.02). Emotional (*p* = 0.01), social (*p* = 0.02), and spiritual (*p* = 0.004) subscales of LASA QOL also showed statistically significant improvements at week 5. There were positive trends toward improvements in stress, sleep and resiliency. To account for potential bias associated with study drop out, analysis was performed comparing 12 patients with end of study 12-week measures to their baseline scores. These findings were consistent with the overall analysis with improvements in anxiety, stress, sleep, fatigue, QOL and resiliency (Appendix A).

**Table 5.** Patient reported outcomes (patients)

Concept Measured	Baseline (*n =* 33)	Week 5 (*n =* 19/20)	Week 9 (*n =* 13)	Week 12 (*n =* 12)
	Median (range)	Median (range)	Median (range)	Median (range)
Anxiety (GAD-7)	7.0 (0,21)	3.5 (0,10)	4.0 (0,12)	3.0 (0,8)*
Stress (PSS)	21.0 (13,27)	19.5 (15,25)	20.0 (14,24)	18.0 (13–22)
Sleep (ISI)	8.0 (0,21)	7.0 (1,18)	7.0 (0,12)	6.0 (0,18)
Quality of Life (LASA)	34.0 (18,53)	37.5 (11,59)*	38.0 (19–53)	39.0 (10–52)
Mental well-being	7.0 (4,9)	7.0 (3–10)	7.0 (4–9)	7.0 (2–10)
Physical well-being	5.0 (0,8)	5.5 (2–10)	5.0 (2–8)	6.0 (1–9)
Emotional well-being	6.0 (1,10)	7.0 (2–10)*	7.0 (3–9)	7.0 (2–9)
Social well-being	5.0 (0,8)	7.0 (1–9)*	5.0 (2–9)	6.0 (1–9)
Spiritual well-being	6.0 (2–9)	7.5 (1–10)*	8.0 (2–9)	7.5 (1–10)
Resiliency(Resiliency Scale)	64.0 (32,91)	73.0 (53,87)*	78.0 (42,94)	71.0 (20,86)
Fatigue(PROMIS-Fatigue)	15.0 (4,20)	12.0 (6,20)	12.0 (4,19)	11.5 (4–17)*

* Significant change from baseline *p* ≤ 0.05.

Caregivers also demonstrated a trend toward improvements in distress, anxiety, and fatigue. There was a significant improvement in stress and QOL at week 5 (*p* = 0.03, 0.03). Details of the effectiveness measures for caregivers are reported in Appendix B.

### Qualitative outcomes

Interviews were conducted with 10 participants: 2 patient/caregiver dyads and 6 patients alone. The interviewees had an age range of 46–72 and included 9 women and 1 man. Content analysis revealed four themes: (1) Easy to Use; (2) Learning Key Principles; (3) Practice is Essential; and (4) Examples of Benefits. Representative quotes for each theme are provided in Table 6. Each of the themes is described below.

**Table 6.** Semi-structured interview quotes

Theme	Select Participant Quotes (Transcript #)
**Easy to Use**	“The daily practices I could embrace and do, [they were] not hard or difficult, [rather], easy and straight forward.” (T4)
	“I guess the best thing that I liked about the program was that I didn’t have to figure it out, it was self-explanatory. (T3)
	“I thought [the speaker in the modules] was really easy to listen to and interesting to listen to … it was very comfortable … it felt like he was more talking right to us. I thought he did a really good job as a presenter.” (T7)
	“In the wintertime it was really good for me because I couldn’t walk [outside]. So it was something that I had to look forward to even when I was feeling bad, if I was sick or throwing up I could still practice it because I didn’t have to … get up, I could read it and reinforce it through reading it.” (T3)
	“I think one could really tell that they (the facilitators) were invested, and that they genuinely cared about helping us to better be able to gauge and work within our emotional state during a really stressful time. I think having the continuity of having the same two people throughout it was helpful.” (T2)
	“Sometimes you were behind on things, and you had a choice of whether to just give up and not do any more or try to catch up, and when you know you have a video visit coming up to talk about it, then I think it gives you the impetus to catch up instead of give up.” (T6)
**Learning Key Principles**	“Instead of being full of that frustrated energy … stopping in the moment and just saying okay, let’s be present for a second and throw gratefulness at this and see where it gets us. That really does help.” (T2)
	“I think as a caregiver … you are trying to get a bunch of … background things done so things are more seamless, and relaxing for [the patient]. But, in doing so … just also reminding myself that, to be cognizant and to be present with him, and [notice] how is he doing? Is there something we could do slower together?” (T2)
	“I felt like it was like pulling up to a gas station but instead of gas you’d get filled up on positivity instead … it was a great infusion of a positive energy fill- up.” (T2)
	“I’ve made that gratitude mine by weaving it into something that I do every day … I step outside, and I just feel that fresh air and I take that time to be grateful for the day … [and reflect on] we are very fortunate to have another day.” (T2)
	“Cancer is cancer. You can fight it, but you very seldom beat it, and I guess I was just blessed because I beat it twice. I am resilient … and a lot of people are not here to talk about it, and … don’t have the support system I have, and…aren’t given the medical support I had … and if you look at it that way you can be grateful for where you are because you could be somewhere less.” (T3)
	“I think [forgiveness is the] hardest to do … I don’t know how much forgiveness I have.” (T6)
**Practice is Essential**	“… recalling those things daily helps to reinforce rather than wait for some magic time to read about them again. I think there are certain elements of that which can be reinforced weekly or daily.” (T7)
	“I need to practice that, to build that muscle, I have to do it more often and that does take time … the great thing is I can go back to it, the book is next to my bed, I brought it on vacation … it was with me because it is important to me.” (T5)
**Examples of Benefit**	“I am a dramatically changed person.” (T4)
	“It taught me how to be more resilient … It may be that you can do something that you thought you couldn’t do, and even though you didn’t want to do it, you did it anyway, and it builds a strength and a character that you didn’t know you had.” (T3)
	“It will teach you how to fight back and how to take back your life as much as you possibly can and not to live in what you can’t do, but to look forward to what you can do.” (T3)
	“… it gave us another opportunity to … process and talk through things and get another person’s perspective. So, I think it helped reinforce the study’s principles and it was also an additional opportunity for the two of us to chat about them. It had benefits beyond the call, it had benefits to the processing that we had during that call then would continue once we were off the call.” (T2)

#### Easy to use

Participant responses confirmed that the program is acceptable and feasible for both caregivers and patients with advanced cancer. They described the program as easy to use, flexible, and felt that the content was appropriate. Participants also reported that the online and remote delivery format was easy to use. Key factors in satisfaction included the ability to view the modules on their own time; the use of short video clips; and that the speaker was engaging with the use of stories and analogies that made the concepts easy to understand. Participants appreciated the convenience of viewing modules online without need for travel to in-person sessions in inclement weather or when not feeling well. Video conferencing was an adequate, convenient delivery mechanism for the facilitated sessions, but some stated that given the choice, they would have preferred in-person visits.

#### Learning key principles

Participants identified and described key learnings and benefits derived from the context of the program as well as content that was more challenging. Keeping a positive attitude, gratitude and mindfulness were the most mentioned strategies. Participants emphasized that the program allowed them to have more control over what they focused on and promoted their ability to choose to focus on the positive.

Gratitude was the resilience principle that most resonated with participants and was the easiest to incorporate into their daily lives. Inherent in their description of their use of gratitude was the ability to focus on the positive. Participants appreciated the recommendation to start the day with a gratitude practice and “weaving it into something” they already do every day. Participants also indicated that practicing gratitude helped them to reframe experiences.

Many participants shared that the program enhanced their understanding of mindfulness practices, and their ability to pull themselves into the present moment and practice awareness which improved their ability to manage the frustrations of the illness process and treatments, and from the caregiver’s perspective, to be more present with their loved one and more cognizant of their emotional needs.

Forgiveness is the resilience principal participants found most challenging to implement. One person stated, “I don’t know how much forgiveness I have.” Participants described using forgiveness to achieve a positive outcome when managing a challenging situation.

#### Practice is essential

Participants described the need to be purposeful about practicing presence and the resilience principles of the RLP on a regular basis. They described specific strategies they used to promote memory and use of the resilience principles, such as reviewing content, re-watching videos, summarizing key points in their own key words, or placing a sticky note with the word “gratitude” or “meditate” in a frequently visited location in their home. They described the desire to have reminders after the completion of the program to prompt them to continue their practice, such as telephone calls, daily texts, emails, or ongoing access to related videos or podcasts.

Practice with a partner was a helpful strategy described by those who participated in the program with their caregiver. It prompted discussion, allowed them to identify shared feelings, and aided in identifying methods to implement the principles together. Some patients who did not have a caregiver participate indicated they preferred doing it alone, primarily because the conversations were too personal. One caregiver stated that they would not have likely participated if their loved one was not interested but were glad that they did.

#### Examples of benefit

The RLP program was described as having a significant impact on participants’ lives. One participant stated it had a dramatic, profound impact on them and that they felt like a “changed person.” Another stated that while they did not always find it easy to keep up with the practices, they learned the importance of sticking with it, and that, in and of itself, taught them how to be resilient and realize that you can accomplish something you thought you were not able to. Others stated that some of the content of the program was not new to them; however, it was helpful to have the reinforcement of the benefits of the practices, to be aware of the science behind the program, and to learn new, practical ways to practice the principles and incorporate them into one’s daily life. The program helped participants reframe stressful events, handle the stress differently, and be more resilient.

All participants indicated that they would recommend the program to others in similar situations. Rationale included that it would help others at a challenging time in their life when many changes are happening to “fight back,” and “take back [their] life,” and not to focus on what they can’t do, but what they can. One participant indicated that the program may be more beneficial at the survivor stage when an individual is not as overwhelmed with the treatment process.

## Discussion

This single arm pilot study demonstrated that delivery of the mindfulness-based RLP via online modules and 4 facilitated video telehealth sessions is feasible and acceptable for patients with advanced cancer and their caregivers. The majority of participants participated in at least 3 of the 4 remote video telehealth sessions. Participants found the virtual component of the program easy to use and appreciated the ability to engage with the online program and video sessions from their home. There was a statistically significant improvement in patient anxiety and fatigue at week 12, as well as improvements in QOL (including emotional, social, and spiritual well-being). Positive trends were also found for stress, resiliency, and sleep. Overall, participants found benefit from the program in that it provided them with skills to practice present moment awareness, gratitude, and focus on the positive. Participants reported that using the program helped decrease their stress and would recommend the program to others in a similar situation.

Individuals living with advanced cancer experience significant psychosocial distress related to uncertainty about prognosis, fear of disease progression, as well as interference with defining life roles. Their caregivers in turn suffer from significant burden and distress. There is continued need to develop interventions and models of care that can support the unique needs of this population of patients and caregivers. Early integrated palliative care (specialty palliative care within 8 weeks of diagnosis of an advanced cancer) has been shown to improve quality of life and mood in patients living with metastatic cancer (Spitzer et al. 2006; Temel et al. 2010). However, there is a shortage of palliative care resources and clinicians compared to the growing population of patients living with advanced cancer (Kamal et al. 2017). A major component of palliative care is support of emotional well-being and coping. A secondary analysis examining the components of a palliative care visit for patients with advanced cancer demonstrated that while nearly 75% of visits addressed symptom management a close second was the coping support (64.2%). In addition, patients who had a higher proportion of visits that addressed coping experienced improved quality of life and depression symptoms (Hoerger et al. 2018). Cognitive coping strategies including mindfulness and gratitude are examples of positive coping strategies encouraged by palliative care clinicians (Greer et al. 2018). These concepts are the basis of the RLP. Given the shortage of palliative care clinicians compared to the growing population of individuals living with advanced cancer (Kamal et al. 2017), the RLP may provide a way to augment services provided by the palliative care team to support adaptive coping in these patients and caregivers.

An additional important finding from the study was the feasibility and acceptability of providing the intervention in a fully remote fashion. Participants indicated they found the program to be easy to navigate, felt comfortable interacting remotely with the study team, and appreciated the convenience of the remote delivery format; however, some indicated that if given the choice, they would have preferred in-person sessions. This would suggest that providing personalized options for delivery format would be advisable in future studies.

This study also demonstrated the ability to have caregivers participate in the intervention with the patient as they attended video sessions together. Cancer caregivers can suffer from a multidimensional burden as they are expected to provide substantial care for their partner or loved one (Applebaum and Breitbart 2013). There is increasing emphasis on the importance of developing psychosocial interventions that support both patient and caregivers (Mollica et al. 2022). Results from this study suggest that some caregivers are willing to participate with the patient; those who completed the intervention found benefit in the outcomes of decreased stress and a positive impact on their relationship. This is consistent with findings of other mindfulness-based intervention studies that included patients with metastatic disease and their caregivers (Milbury et al. 2020a, 2020b).

Finally, the study demonstrated a positive trend toward improvement in fatigue in the patient group. Fatigue is multifactorial and difficult to treat in this population. It is known that fatigue has affective and cognitive components, and this may at least partially explain the observed changes in self-reported fatigue. Further research could be done to explore the effect of the RLP on fatigue.

### Limitations of the study

This study is limited by a small sample size and a relatively high attrition (33 completed baseline questionnaires; 12 completed 12-week measures). A high attrition rate is common in studies of patients with advanced cancer, often reported around 20% (Cheville et al. 2019; Temel et al. 2020). These findings may support the need to enroll patients earlier in disease trajectory or have an option for a more concise intervention. Moreover, the numbers of caregivers who participated was small and there was significant loss of survey response in this group; therefore, it is difficult to conclude the true effect of the RLP on caregivers. However, there was a trend toward positive improvement in the small number who completed the surveys and participated in the qualitative interviews. Based on anecdotal feedback to study staff one of the main barriers to caregiver participation in the intervention was being too busy or not having enough time. Therefore, it may be necessary to develop a more concise, less time-intensive options for caregivers. Additionally, it would be warranted to collect more specific feedback from caregivers in subsequent studies to better understand the barriers. Furthermore, it should be noted that the RLP did not focus on specific content related to caregiving and it may be that some caregivers would benefit more from a caregiver specific intervention or may prefer to receive support individually.

There are intervention design factors that may limit scalability of the intervention. For example, the interventionists included a nurse scientist (RN/PhD), clinical nurse specialist (CNS, DNP), and physician (MD). Given the ongoing nursing shortage and expense of physician time, this may not be replicable in the practice setting. An option to make the RLP more cost-effective would be to utilize community health workers (CHW) or health coaches as interventionists. A CHW is a trained health worker who may not have a clinical background. CHW engagement with advanced cancer patients has been shown to increase the use of palliative care and hospice and improve mental and emotional health (Patel and Kapphahn 2022). In addition, 1:1 video sessions may not be scalable; therefore, studies assessing outcomes of a group intervention are warranted. Group psychosocial interventions in patients with advanced cancer have been shown to be acceptable and effective in improving mood and quality of life (Rummans et al. 2006; Breitbart et al. 2015, 2018).

The Resilience Scale used in this study was developed by members of the research team based on 10 conceptualized resiliency factors that were essential to individuals being able to thrive following significant stressful events (Southwick et al. 2014, 2015). There has been no formal psychometric evaluation of this scale published.

Another limitation is that most participants were white females, which is somewhat reflective of the demographics of the recruitment site, as well as the caregiver population (predominantly women). Next steps would involve a multisite trial to increase diversity. However, a strength is that the study had representation from multiple tumor types.

## Conclusion

In summary, the RLP was feasible and acceptable in a population of patients with advanced cancer and their caregivers. Participants experienced improvements in anxiety, QOL, and fatigue. Positive trends were identified in stress, sleep, and resiliency.

Participants indicated the program enhanced their ability to focus on the positive and cope with their experience of illness. Next steps include larger, multisite trials with a more diverse population, testing outcomes of more scalable intervention designs. Additionally, individuals’ needs and experiences with advanced cancer are personal and unique. Although overall patients and caregivers found the experience of the RLP positive and beneficial, some would have preferred to focus more on specific concepts, such as forgiveness. Adapting the RLP to a more individualized and targeted program would allow more flexibility to adapt the unique needs of each participant.

## Supporting information

Chesak et al. supplementary material Chesak et al. supplementary material

## Supplementary material

The supplementary material for this article can be found at https://doi.org/10.1017/S1478951524002128.
