# Supplementary material for: Resilient living program for patients with advanced cancer and their caregivers
Source: Palliat Support Care. 2025 Mar 14;23:e75. doi: 10.1017/S1478951524002128 (PMC13166411; doi:10.1017/S1478951524002128)
Supplement: Chesak et al. supplementary material [file S1478951524002128sup001.docx]

Appendix A. Changes in Outcomes Measures Over Time among patients with data at baseline and 12 week (Patients)

| Concept Measured | Baseline (*n*=12)  median (range) | Week 12  (*n*=12)  median (range) |
| --- | --- | --- |
| Anxiety (GAD-7) | 5.5 (0,11) | 3 (0, 8) |
| Stress (PSS) | 20.5 (13,23) | 18 (13, 22) |
| Sleep (ISI) | 9.5 (0, 17) | 6 (0, 18) |
| Quality of Life | 34 (18, 53) | 39 (10, 52) |
| Resiliency (Resiliency Scale) | 66.5 (32, 91) | 71 (20, 86) |
| Fatigue (PROMIS-Fatigue) | 15 (4, 20) | 11.5 (4-17) |

Appendix B

Changes in Outcomes Measures Over Time (Caregivers)

| Concept Measured | Baseline (*n*=15)  median (range) | Week 5  (*n*=6)  median (range) | Week 9  (*n*=5)  median (range) | Week 12  (*n*=4)  median (range) |
| --- | --- | --- | --- | --- |
| Anxiety (GAD-7) | 7.0 (0,18) | 6 (3,9) | 3 (0, 7) | 5 (0, 12) |
| Stress (PSS) | 22 (16,32) | 21 (17,22) | 18 (16,21) | 21 (17-23) |
| Sleep (ISI) | 8 (0, 18) | 5 (0, 9) | 7 (0,11) | 6 (0, 11) |
| Quality of Life | 39 (7, 54) | 39 (29,50)* | 38 (33-52) | 38 (19-48) |
| Resiliency (Resiliency Scale) | 69 (43, 88) | 63 (47, 90) | 76 (52, 84) | 74.5 (57, 84) |
| Fatigue (PROMIS-Fatigue) | 10 (4, 20) | 8.5 (5,14) | 8 (4,8) | 8.5 (4-13) |
